# Attention-Deficit/Hyperactivity Disorder Remission Is Linked to Better Neurophysiological Error Detection and Attention-Vigilance Processes

**DOI:** 10.1016/j.biopsych.2016.06.021

**Published:** 2016-12-15

**Authors:** Giorgia Michelini, Glenn L. Kitsune, Celeste H.M. Cheung, Daniel Brandeis, Tobias Banaschewski, Philip Asherson, Gráinne McLoughlin, Jonna Kuntsi

**Affiliations:** a MRC Social, Genetic and Developmental Psychiatry Centre, London, United Kingdom; b Department of Psychological Medicine, London, United Kingdom; c Department of Psychology, Institute of Psychiatry, Psychology and Neuroscience, King’s College London, London, United Kingdom; d Department of Psychological Sciences, Centre for Brain and Cognitive Development, Birkbeck, University of London, London, United Kingdom; e Department of Child and Adolescent Psychiatry and Psychotherapy , Central Institute of Mental Health, Medical Faculty Mannheim/Heidelberg University, Mannheim, Germany; f Department of Child and Adolescent Psychiatry and Psychotherapy, Psychiatric Hospital, Zurich, Switzerland; g Center for Integrative Human Physiology, Zurich, Switzerland; h Neuroscience Center Zurich, University of Zurich, Zurich, Switzerland

**Keywords:** ADHD, Cognitive impairments, EEG, Event-related potentials, Persistence, Remission

## Abstract

**Background:**

The processes underlying persistence and remission of attention-deficit/hyperactivity disorder (ADHD) are poorly understood. We examined whether cognitive and neurophysiological impairments on a performance-monitoring task distinguish between ADHD persisters and remitters.

**Methods:**

On average 6 years after initial assessment, 110 adolescents and young adults with childhood ADHD (87 persisters, 23 remitters) and 169 age-matched control participants were compared on cognitive-performance measures and event-related potentials of conflict monitoring (N2) and error processing (error-related negativity and positivity) from an arrow flanker task with low-conflict and high-conflict conditions. ADHD outcome was examined with parent-reported symptoms and functional impairment measures using a categorical (DSM-IV) and a dimensional approach.

**Results:**

ADHD persisters were impaired compared with controls on all cognitive-performance and event-related potential measures (all *p* < .05). ADHD remitters differed from persisters and were indistinguishable from control participants on the number of congruent (low-conflict) errors, reaction time variability, error-related negativity, and error-related positivity (all *p* ≤ .05). Remitters did not differ significantly from the other groups on incongruent (high-conflict) errors, mean reaction time, and N2. In dimensional analyses on all participants with childhood ADHD, ADHD symptoms and functional impairment at follow-up were significantly correlated with congruent errors, reaction time variability, and error-related positivity (*r* = .19–.23, *p* ≤ .05).

**Conclusions:**

Cognitive and neurophysiological measures of attention-vigilance and error detection distinguished ADHD remitters from persisters. These results extend our previous findings with other tasks and indicate that such measures are markers of remission and candidates for the development of nonpharmacological interventions.

The identification of cognitive and neural processes underlying the trajectories of persistence and recovery from childhood-onset disorders during the transition to adulthood has the potential to prevent negative long-term outcomes (1, 2). Attention-deficit/hyperactivity disorder (ADHD) is a neurodevelopmental disorder affecting 5%–6% of children and adolescents worldwide (3, 4). ADHD often persists into adulthood, where the prevalence rate is around 2%–3% (5), with severe impacts on many aspects of individuals’ lives (6, 7). Although in a proportion of cases ADHD symptoms reduce to subclinical levels from childhood to adulthood (8), little is known about the compensatory processes and enduring deficits of ADHD.

It has been proposed that the cognitive processes associated with persistence of ADHD across development may be separate from those linked to the remission of the disorder (9). However, empirical data to date are inconsistent with regard to the exact pattern of cognitive impairments that distinguish ADHD remitters from persisters. Whereas some studies comparing ADHD remitters and persisters have linked remission to better executive function performance (1, 10), other studies have found no differences between ADHD remitters and persisters in adolescence and adulthood on measures of executive functions (11, 12, 13, 14, 15).

The assessment of neurocognitive processes using cognitive and brain activity data may allow a deeper understanding of the developmental trajectories of ADHD. Our recent investigation of adolescents and young adults with childhood ADHD assessed on a range of cognitive, event-related potential (ERP), and electroencephalography (EEG) measures found that ADHD remitters differed from persisters, but not from control participants, on preparation-vigilance measures (reaction time variability [RTV], omission errors, ERP activity of response preparation, and delta and theta activity) and actigraphic data on movement. Executive-function processes of inhibition and working memory (commission errors, digit span backward, and ERP activity of inhibitory control), instead, were not sensitive to ADHD persistence or remission, as ADHD remitters showed an intermediate pattern between persisters and control participants, without significant differences from either group (14). Further combined investigations of cognitive and neurophysiological data may aid our understanding of the mechanisms underlying ADHD remission and persistence.

Neurocognitive impairments in ADHD include deficits in performance monitoring, an essential cognitive ability in goal-directed behavior to monitor ongoing performance and to adjust response selection (16, 17, 18). The investigation of performance-monitoring impairments with ERP parameters, including the N2 and the error-related negativity (ERN) and positivity (Pe), in individuals with ADHD may provide new information to elucidate the neurocognitive pathways of remission. The N2 is a frontocentral stimulus-locked negative deflection mostly observed 200–400 ms after the presentation of stimuli inducing high conflict (such as incongruent stimuli) and when a correct response is made (17, 19). This ERP reflects a conflict-monitoring process, as it results from the conflict arising from two competing responses and evaluation of the correct response (19). When a participant makes an error, the ERN, a frontocentral response-locked negative deflection at around 0–150 ms is observed, followed by the Pe, a centroparietal positive enhancement at around 200–400 ms after response (20, 21, 22). The ERN is thought to reflect unconscious activity of a generic response-monitoring system immediately after a mistake is made, whereas the Pe is thought to represent conscious error processing to adjust response strategy (23).

In ADHD, N2 attenuation in the flanker task has been reported in children and adults with ADHD (24, 25, 26), although two smaller studies failed to replicate this finding (27, 28). With regard to ERN and Pe attenuation in ADHD, a recent meta-analysis found an overall ERN attenuation in performance-monitoring tasks (29). Pe attenuations in ADHD samples were significant in Go/NoGo tasks, but not flanker tasks. Yet, data on these ERPs in individuals with ADHD are overall limited, and study samples have remained small. Furthermore, studies have not, to date, investigated the association between neurophysiological performance monitoring and ADHD persistence and remission. One recent study showed that ERN and Pe deficits may be improved with motivational incentives or methylphenidate medication in ADHD groups (30), suggesting malleability of the error-processing impairments in ADHD.

In the present study, we aimed to extend our recent findings (14) by investigating cognitive and neurophysiological impairments from a performance monitoring task in adolescents and young adults with persistent and remitted ADHD. We examined ADHD outcome with parent-reported symptoms and functional impairment measures using both a categorical (DSM-IV) and a dimensional approach. Based on our previous results and evidence of potentially malleable neurophysiological error processing, we predicted that cognitive measures underlying nonexecutive processes and ERPs of error processing (ERN/Pe) would distinguish between ADHD persisters and remitters and would represent markers of remission. We further predicted that cognitive indices of executive control would not vary with persistence or remission of ADHD. No formal predictions were made for ERP measures of conflict monitoring (N2), owing to absence of any evidence suggesting a possible association with remission or persistence of ADHD.

## Methods and Materials

### Sample

The sample consists of 279 participants, who were followed up on average 5.8 years (SD = 1.1) after initial assessments: 110 had a diagnosis of DSM-IV combined-type ADHD in childhood (10 sibling pairs and 90 singletons) and 169 were control participants (76 sibling pairs and 17 singletons) (14, 31). Participants with ADHD were initially recruited from specialized ADHD clinics (32) and control participants from schools in the United Kingdom. Information on any diagnosed neurodevelopmental and psychiatric conditions and medication use were collected through neuropsychiatric screening. Exclusion criteria at both assessments included IQ < 70, autism, epilepsy, brain disorders, and any genetic or medical disorder associated with externalizing behaviors that might mimic ADHD. Other comorbidities were not excluded in order to have an ADHD sample that is representative of the clinical population. At follow-up, we excluded six control participants who met DSM-IV ADHD criteria based on the parent-reported Barkley Informant Rating Scale (33) and six participants with ADHD who had missing parent ratings of clinical impairments. Two participants with childhood ADHD, who did not meet ADHD symptom criteria but met clinical levels of impairment at follow-up, were also excluded to minimize heterogeneity in the sample.

Among those with childhood ADHD, 87 (79%) continued to meet clinical (DSM-IV) levels of ADHD symptoms and impairment (ADHD “persisters”), whereas 23 (21%) were below the clinical cut-off (ADHD “remitters”) (31). Among ADHD remitters, 14 displayed ≥5 items on either the inattention or hyperactivity/impulsivity symptom domains, but they did not show functional impairment. ADHD persisters, remitters, and control participants did not differ in age, but there were significantly more male participants in the remitted group than in the other two groups, with no female participants among ADHD remitters (Table 1). Participants attended a single research session for clinical, IQ, and cognitive-EEG assessments. Almost one-half (47%) of the participants with childhood ADHD were being treated with stimulant medication at follow-up. Those who were on medication scored significantly higher on ADHD symptoms (*F* = 11.34, *p* < .01) and functional impairment (*F* = 5.22, *p* < .01) than those who were not taking medication. However, the proportion of participants on medication did not differ between ADHD persisters and remitters (χ^2^ = 1.95, *p* = .16). A 48-hour ADHD medication-free period was required prior to assessments. Three ADHD persisters (3.4%) were also on antidepressant medication, but for ethical reasons they were not asked to stop taking them. These participants were included in all analyses as their exclusion did not alter the results. Parents of all participants gave informed consent following procedures approved by the London-Surrey Borders Research Ethics Committee (09/H0806/58).

### ADHD Diagnosis

The Diagnostic Interview for ADHD in adults (DIVA) (34) was conducted by trained researchers with parents of the ADHD probands to assess DSM-IV-defined ADHD presence and persistence. Raw scores for inattention and hyperactivity/impulsivity symptoms (range 0–9 for each dimension) were generated for each participant. Evidence of impairment commonly associated with ADHD was assessed with the Barkley’s Functional Impairment Scale (33) during interviews with parents. Each item ranges from 0 (never or rarely) to 3 (very often). Participants were classified as “affected” at follow-up if they scored ≥6 in either the inattention or hyperactivity/impulsivity domains on the DIVA and ≥2 on two or more areas of impairments on the Barkley’s Functional Impairment Scale. We defined ADHD outcome using a categorical definition of persistence based on diagnoses, as well as a dimensional approach based on levels of symptoms of ADHD and impairments measured as continuous traits.

### IQ Assessment

An estimate of IQ was derived with the vocabulary and block design subtests of the Wechsler Abbreviated Scale of Intelligence (35).

### Task

The task was an adaptation of the Eriksen Flanker paradigm designed to increase cognitive load as used in previous studies (24, 25, 36). In each trial, a central black fixation mark was replaced by a target arrow (a black 18-mm equilateral triangle). Participants had to indicate whether this arrow pointed toward the left or right by pressing corresponding response buttons with their left or right index fingers. Two flanker arrows identical in shape and size to the target appeared 22 mm above and below the center of the target arrow 100 ms prior to each target arrow. Both flankers pointed in either the same (congruent) or opposite (incongruent) direction to the target. As such, conflict monitoring is maximal during the incongruent condition. When the target appeared, both target and flankers remained on the screen for a further 150 ms, with a new trial being presented every 1650 ms. Two hundred congruent and 200 incongruent trials were arranged in 10 blocks of 40 trials over 13 minutes. For further details on the task, see the Supplement. Cognitive-performance measures of mean reaction time (MRT), RTV (SD of reaction times), and number of errors (left-right errors occurring when participants chose the wrong left or right response) were calculated separately for congruent and incongruent conditions.

### Electrophysiological Recording and Processing

The EEG was recorded from a 62-channel DC-coupled recording system (extended 10–20 montage), using a 500-Hz sampling rate, impedances under 10 kΩ, and the FCz electrode as the recording reference. The electro-oculograms were recorded from electrodes above and below the left eye and at the outer canthi. EEG data were analyzed using Brain Vision Analyzer 2.0 (Brain Products, Gilching, Germany). Raw EEG recordings were down-sampled to 256 Hz, rereferenced to the average of all electrodes (turning FCz into an active channel), and filtered using Butterworth band-pass filters (0.1–30 Hz, 24 dB/octave). All trials were visually inspected for electrical artifacts or obvious movement, and sections of data containing artifacts were removed manually. Ocular artifacts were identified using the InfoMax independent component analysis algorithm (37). Sections of data containing artifacts exceeding ±100 μV or with a voltage step >50 μV were automatically rejected. Baseline correction was applied using the −300 to −100 ms pretarget (−200 to 0 ms preflanker) interval.

**Figure 1:**
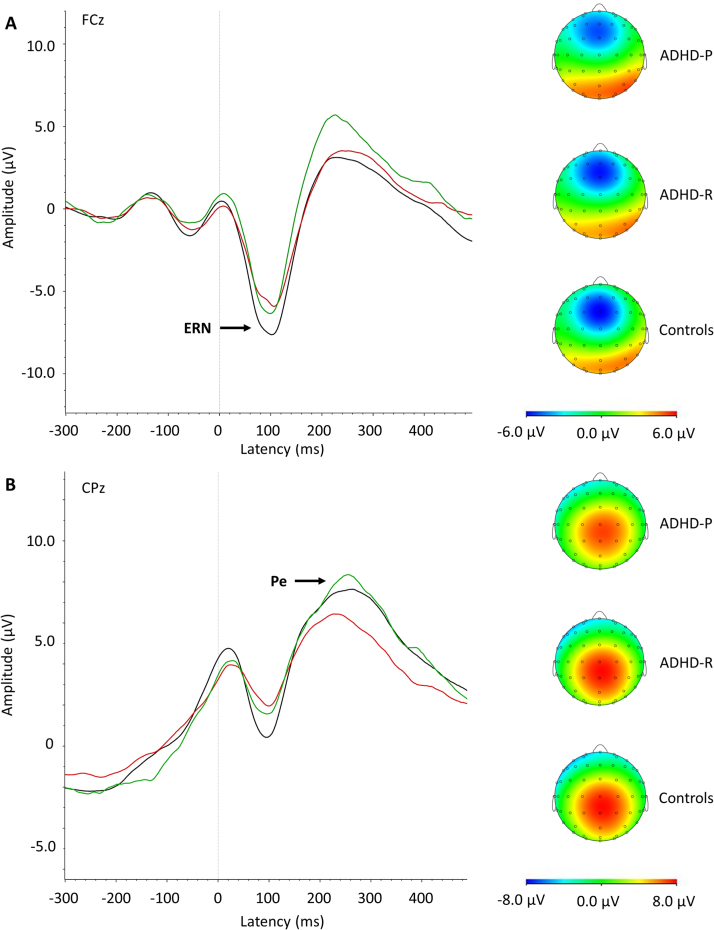
Grand average response-locked event-related potentials of the error-related negativity (ERN) at the FCz electrode between 0 and 150 ms **(A)** and the error-related positivity (Pe) at the CPz electrode between 150 and 450 ms **(B)** after an erroneous response on the incongruent trials for attention-deficit/hyperactivity disorder (ADHD) persisters (ADHD-P, in red), ADHD remitters (ADHD-R, in green), and control participants (Controls, in black), with topographic maps.

**Figure 2:**
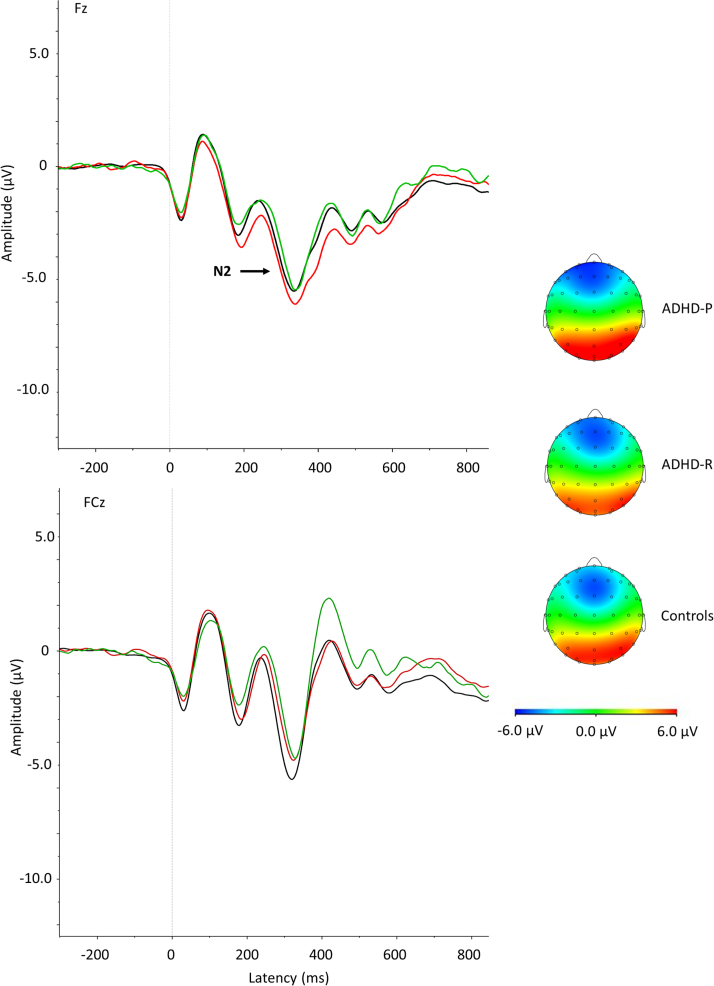
Grand average stimulus-locked event-related potentials of the N2 at the Fz and FCz electrodes between 250 and 450 ms after incongruent stimuli where a correct response was made for attention-deficit/hyperactivity disorder (ADHD) persisters (ADHD-P, in red), ADHD remitters (ADHD-R, in green), and control participants (Controls, in black), with topographic maps.

Analyses of ERPs of performance monitoring were restricted to incongruent trials, as the task used in this study is known to elicit strong N2, ERN, and Pe components in high-conflict, but not in low-conflict, conditions (24, 25, 36). Data were segmented based on 1) stimulus-locked incongruent trials where a correct response was made and 2) response-locked (error-related) incongruent trials where an incorrect response was made. Individual averages were created based on each condition, requiring ≥20 clean segments for each participant. After averaging, the electrodes and latency windows for ERP analyses were selected based on previous studies (23, 24, 25, 38), topographic maps, and the grand averages (Figure 1, Figure 2). The N2 was measured as maximum negative peak at the Fz and FCz electrodes between 250 and 450 ms after target onset. The ERN was defined with respect to the preceding positivity (PNe, –100 to 50 ms) and measured at FCz between 0 and 150 ms. This peak-to-peak measure has proven to be a robust measure of this component (20, 23, 39) and was favored over a peak-to-baseline (maximal amplitude) measure as the former distinguished ADHD from control participants in independent samples using this version of the Eriksen Flanker task (24, 25, 40); it was therefore the ideal candidate in relation to ADHD remission/persistence (for further details see the Supplement). The Pe was measured as maximum positive peak at the CPz electrode between 150 and 450 ms after an erroneous response on incongruent trials.

### Statistical Analyses

For RTV and errors, we tested overall effects of group (ADHD persisters, remitters, control participants), condition (congruent, incongruent), and group by condition interaction using random intercept models in Stata (StataCorp, College Station, TX) to control for genetic relatedness of the sibling pairs in a repeated-measures design. A random intercept model was also run to test the effect of group, scalp site (Fz, FCz) and group by site interaction on the N2. ERN and Pe were analyzed with regression models with dummy variables to identify overall group effects, controlling for sibling relatedness with the “robust cluster” command in Stata. Age correlated significantly with several of the cognitive-ERP measures (Supplemental Table S1) and was therefore included as a covariate in group analyses. On measures that indicated a group effect, post hoc regressions were performed. The majority of our sample consisted of male participants (80%), and thus primary analyses were performed on the whole sample without accounting for sex differences. As groups were not matched on sex (no female in the sample remitted from ADHD) (Table 1), analyses were rerun with the female participants (15 ADHD persisters and 41 control participants) removed. Cohen’s *d* effect sizes are presented along with means, SDs, and test statistics for the group analyses (Table 2), where 0.20 is considered a small effect, 0.50 a medium effect, and 0.80 a large effect (41). Pearson correlations examined which measures correlated with DIVA ADHD symptom scores and functional impairment in those with a childhood ADHD diagnosis, with age and sex included as covariates.

Because ADHD persisters had lower IQs than remitters did (Table 1) (14), and higher IQ in childhood was associated with ADHD remission at follow-up in this sample (31), all analyses were also rerun controlling for IQ. All cognitive-ERP measures were skewed and log-transformed to normal. Three participants (ADHD persisters) were excluded from the N2 analysis and 39 (13 ADHD persisters [15%], 3 ADHD remitters [13%], 23 control participants [14%]) from the ERN/Pe analysis due to having <20 artifact-free ERP segments, which is similar to previous studies using this paradigm (24, 25), and reflecting a similar exclusion ratio across groups.

## Results

### Group Differences

An overall group effect emerged on all cognitive-performance and ERP measures (Table 2, Figure 1, Figure 2). Post hoc analyses showed that ADHD persisters had significantly higher MRT, RTV, number of errors, enhanced N2 (at Fz, but with a trend for reduction at FCz, pointing to topographic differences, as shown in Supplemental Figure S1) and reduced ERN and Pe compared with control participants, with small-to-large effect sizes. Significant differences between ADHD remitters and persisters emerged on congruent and incongruent RTV, congruent errors, ERN, and Pe with medium-to-large effect sizes. ADHD remitters did not differ from persisters on MRT in either condition, on incongruent errors and N2, with null-to-small effect sizes. ADHD remitters and control participants significantly differed on incongruent RTV, with a medium effect size, and at trend level with small effect sizes for incongruent errors and incongruent MRT.

Controlling for IQ, group effects on MRT in both conditions and N2 at FCz were nonsignificant (Table 2). Differences between remitters and persisters became nonsignificant in incongruent RTV and trends in ERN and Pe. Remitters and control participants differed at trend level in incongruent RTV, but not in incongruent errors. Results for other variables remained unchanged. When repeating the analyses with female participants removed, the difference between ADHD persisters (*n* = 63) and remitters (*n* = 20) became a trend for the ERN and nonsignificant for the Pe. Given the small female sample sizes (*n* = 15; of which only *n* = 11 had data on ERN and Pe) and the discrepancy in the size of male and female groups, sex differences were not directly tested. However, the effect sizes in the male-only sample (*d* = 0.47 for the ERN, *d* = 0.34 for the Pe) were comparable or only slightly reduced compared with those of the full sample. Remitters significantly differed from control participants on incongruent MRT, congruent RTV, and incongruent RTV, but not on incongruent errors. All other results remained unchanged. For further details, see the Supplement.

### Associations With ADHD Symptoms and Impairments

Among those with childhood ADHD (*n* = 110), both ADHD symptoms and impairment at follow-up significantly correlated with the Pe (Table 3). ADHD symptoms also significantly correlated with RTV in both conditions, and functional impairment correlated with congruent errors and at trend level with incongruent RTV and N2 at Fz. When IQ was controlled for, the correlation of ADHD symptoms or impairment with RTV became nonsignificant, and the correlation between functional impairment and congruent errors became a trend (Table 3).

## Discussion

In this first large-scale investigation of cognitive and neurophysiological performance monitoring in adolescents and young adults with ADHD, we found that ADHD remitters had enhanced cognitive processes of attention-vigilance (RTV and congruent errors) and neurophysiological error processing (ERN and Pe) compared with persisters. Attention-vigilance measures and conscious error processing were also associated with the continuum of ADHD symptoms and impairment at follow-up. Conversely, measures of executive control (incongruent errors), speed of processing (MRT), and neurophysiological conflict monitoring (N2) did not distinguish remitters from persisters, and thus they were not sensitive to remission or persistence of the disorder. Processes of attention-vigilance and neurophysiological error processing can be markers of remission from ADHD and may be sensitive to the effects of training or compensatory mechanisms.

RTV, measuring intraindividual variability in reaction time, and number of congruent errors in the low-conflict condition distinguished ADHD remitters from persisters, but not from control participants, and were also correlated with continuous ratings of ADHD symptoms and impairment. Impairments in such measures in the congruent condition of the flanker task may result from lapses in attention and index attention-vigilance processes. Neurophysiological measures of error processing (ERN and Pe) showed the same association with ADHD remission. Conscious error processing (Pe) also correlated with the continuous ADHD symptoms and functional impairments at follow-up. Of note, the group differences observed on this peak-to-peak ERN were likely explained by the voltage change from the PNe to the negative ERN peak (see the Supplement). This measure captures the response-locked oscillatory pattern immediately before and after an error is made and as such may reflect early attentional processes linked to automatic error detection. Conversely, incongruent errors in the high-conflict condition, likely reflecting a failure in executive control, and MRT in left-right responses at every trial, likely measuring speed of processing in this task that induces high cognitive demands, did not distinguish ADHD remitters from persisters. Similarly, neurophysiological conflict monitoring (N2) did not differ between ADHD groups, potentially indicating suboptimal parallel stimulus processing regardless of remission or persistence (17, 42). Remitters also showed lower RTV in the incongruent condition compared with persisters but were still impaired when compared with control participants. Given the higher levels of executive control elicited in the incongruent condition, this could result from joint influences of both attention-vigilance and executive processes. Therefore, RTV in the incongruent condition may be less sensitive to remission than it is in the congruent condition.

Primary analyses did not control for IQ, as lower-mean IQ in ADHD samples represents one of multiple cognitive processes underlying ADHD pathophysiology (43, 44), and the etiological influences shared between ADHD and IQ are largely separate from those shared with other cognitive impairments (45, 46, 47). Thus, by removing IQ effects when investigating the relationship between ADHD and cognitive-ERP variables, one may also control for features of ADHD related to IQ (48, 49). In this sample, ADHD remission was associated with higher IQ measured both in childhood and at follow-up (14, 31). As such, it may be that higher IQ represents a potential compensatory mechanism. To test the association between cognitive-ERP measures and remission or persistence beyond the influence of IQ, we also repeated the analyses covarying for IQ. When controlling for IQ, overall group differences for MRT were no longer significant, suggesting that group differences on this measure may reflect ADHD impairments related to IQ. Moreover, remitters were more similar to persisters in some markers of remission (RTV, ERN, and Pe) when removing the IQ effects. This further points to an association between IQ and better cognitive-neurophysiological profiles in ADHD remitters.

The present study extends the findings in our previous investigation that used a cued continuous performance test (CPT-OX), a four-choice reaction time task, and Wechsler Abbreviated Scale of Intelligence measures of IQ and digit span (14). Attention-vigilance and error detection showed a similar pattern to that found in our previous analyses for preparation-vigilance measures (RTV, omission errors, ERP activity of response preparation, and delta and theta activity), whereas executive control (measured by incongruent errors), speed of processing (MRT for left-right responses), and conflict monitoring (N2) did not distinguish remitters from persisters, which is similar to measures of inhibition and working memory in our previous investigation (14). ADHD remitters showed an intermediate pattern between persisters and control participants on this latter group of measures: they showed no significant differences from either group on the N2, but there were trend-level differences from control participants on incongruent errors and MRT, suggesting that the latter two measures may potentially represent markers of enduring deficits. Our findings align with four recent studies reporting no differences between ADHD remitters and persisters in executive control (11, 12, 13, 14), but not with two earlier studies that suggested a link from ADHD remission to better executive function (1, 10). More broadly, our findings are in line with evidence for a separation of ADHD neurocognitive impairments into bottom-up and top-down processes supported by genetically sensitive studies (32, 50). Our results are also consistent with reports of ADHD-sensitive improvement following rewards in RTV and ERPs of error processing (30, 51, 52), suggesting that such processes are malleable and may improve with the additional allocation of cognitive arousal and motivational incentives in ADHD samples. Future studies may further characterize the relationship between ADHD outcome and performance monitoring processes by using tasks with different ratios of congruent and incongruent trials, which may produce stronger enhancement of conflict processes (53), potentially coupled with single-trial measures to examine trial-to-trial adjustments (54).

A limitation of this study is that, despite the large sample size, the low ADHD remission rate at follow-up resulted in a relatively small group of remitters. Therefore, we could not rule out the possibility that some nonsignificant differences between remitters and other groups could be due to low power. However, we observed medium-to-large effect sizes (*d* = 0.44–0.75) between persisters and remitters in measures representing markers of remission, but small or negligible effect sizes (*d* = 0.02–0.28) in measures not sensitive to ADHD outcome at follow-up, suggesting this study had sufficient power to detect the major correlates of remission with the current sample sizes. Furthermore, when we repeated the analyses for male participants only, differences between remitters and persisters in the ERN and Pe were reduced. However, the small sample of female participants did not allow a direct examination of sex differences. Future studies that include a higher number of female participants are needed to further investigate these processes also in females. Finally, our sample included young adults as well as adolescents, who are still undergoing rapid cortical maturation. Although we controlled for age in all analyses, future follow-up assessments with participants having reached adulthood and when more ADHD participants may have remitted could clarify matters further.

Overall, we report that attention-vigilance and neurophysiological error processes were impaired in ADHD persisters but not in remitters and may be sensitive to compensatory mechanisms in those who remit from the disorder. These processes may be targets for nonpharmacological interventions or behavioral training aimed at alleviating some of the long-term outcomes of ADHD. Conversely, cognitive measures of executive control, speed of processing, and conflict monitoring were not sensitive to ADHD remission/persistence. Considering the importance of using a broad range of cognitive and neural measures in investigating the mechanisms underlying neurodevelopmental disorders (2), our cognitive and neurophysiological investigation provides an improved understanding of the trajectories to ADHD remission and persistence. Future studies should aim to investigate the neural sources and neurobiological mechanisms underlying these markers of remission in order to pave the way toward the development of new interventions aimed at stimulating processes that are sensitive to remission to reduce severe long-term outcomes of the disorder.

## Figures and Tables

**Table 1:** Sample Demographics Divided by Group, With Test for Group Differences

	ADHD-P	ADHD-R	Ctrl	*p*	ADHD-P vs. Ctrl	ADHD-P vs. ADHD-R	ADHD-R vs Ctrl
					*p*	*p*	*p*
Sex (M:F)	72:15	23:0	129:40	.02^a^	.24	.03^a^	<.01^b^
Age, Years, Mean ± SD	18.27 ± 3.03	18.89 ± 3.06	18.77 ± 2.19	.15	—	—	—
IQ, Mean ± SD	96.20 ± 15.33	104.57 ± 13.63	109.98 ± 12.42	<.01^b^	<.01^b^	.02^a^	.10

Group differences on sex were tested via chi-square test; group differences on age and IQ were tested with regression models. Group differences in sex, age, and IQ were previously reported in another paper on this sample (14).

ADHD-P, attention-deficit/hyperactivity disorder persisters; ADHD-R, attention-deficit/hyperactivity disorder remitters; Ctrl, control group; F, female, M, male.

a *p* < .05.

b *p* < .01.

**Table 2:** Descriptive Statistics and Group Comparison on Cognitive-Performance and ERP Measures

					Group Comparison							Covarying IQ					
	ADHD-P	ADHD-R	Ctrl		ADHD-P vs. Ctrl		ADHD-P vs. ADHD-R		ADHD-R vs. Ctrl		*p*	ADHD-P vs. Ctrl		ADHD-P vs. ADHD-R		ADHD-R vs. Ctrl	
	Mean ± SD	Mean ± SD	Mean ± SD	*p*	*d*	*p*	*d*	*p*	*d*	*p*		*d*	*p*	*d*	*p*	*d*	*p*
Performance																	
Congruent errors	10.89 ± 17.26	4.00 ± 3.85	4.14 ± 8.31	<.01^a^	.83^b^	<.01^a^	.75^c^	<.01^a^	.04	.95	<.01^a^	.55^c^	<.01^a^	.60^c^	.01^a^	.09	.89
Incongruent errors	57.87 ± 20.08	56.22 ± 20.75	48.87 ± 18.02	<.01^a^	.53^c^	<.01^a^	.06	.86	.46	.06^d^	<.01^a^	.32	.01^a^	.06	.98	.37	.11
Congruent MRT (ms)	355.82 ± 60.39	339.58± 38.99	336.25 ± 33.28	<.01^a^	.41	<.01^a^	.28	.23	.11	.63	.28	—	—	—	—	—	—
Incongruent MRT (ms)	449.87 ± 56.16	441.94 ± 33.44	431.68 ± 40.75	<.01^a^	.40	<.01^a^	.07	.73	.35	.07^d^	.44	—	—	—	—	—	—
Congruent RTV (ms)	114.26 ± 65.70	83.19 ± 28.22	76.24 ± 21.67	<.01^a^	1.00^b^	<.01^a^	.61^c^	<.01^a^	.35	.11	<.01^a^	.60^c^	<.01^a^	.42	.04^e^	.14	.25
Incongruent RTV (ms)	119.31 ± 80.64	88.18 ± 32.91	76.12 ± 22.84	<.01^a^	.97^b^	<.01^a^	.47	.04^e^	.50^c^	.02^e^	<.01^a^	.55^c^	<.01^a^	.24	.18	.30	.08^d^
ERPs																	
N2 at Fz (µV)	−7.23 ± 3.69	−6.91 ± 3.61	−6.57 ± 3.27	.02^e^	.30	.03^e^	.02	.91	.29	.19	.03	.25	.02^e^	.01	.88	.26	.20
N2 at FCz (µV)	−5.8 ± 3.74	−6.26 ± 3.57	−6.92 ± 3.81	.07^d^	.26	.08^d^	.18	.53	.08	.82	.11	—	—	—	—	—	—
ERN at FCz (µV)	7.78 ± 3.37	9.64 ± 4.11	10.08 ± 4.51	<.01^a^	.55^c^	<.01^a^	.52^c^	.05^e^	.06	.86	<.01^a^	.37	<.01^a^	.39	.09^d^	.01	.98
Pe at CPz (µV)	9.36 ± 4.23	10.96 ± 4.06	11.31 ± 4.27	<.01^a^	.44	<.01^a^	.44	.05^e^	.02	.88	.03^e^	.32	.03^e^	.36	.06^d^	.06	.79

Data on performance measures were available for the full sample (87 ADHD-P, 23 ADHD-R, and 169 control participants); data on the N2 were available for 84 ADHD-P, 23 ADHD-R, and 169 control participants; data on the PNe, ERN, and Pe were available for 74 ADHD-P, 20 ADHD-R, and 146 control participants. Overall effects of group, condition (on cognitive-performance measures), and site (on the N2) and interaction effects were tested with mixed models and reported in Supplemental Table S2. Only group effects were tested on the ERN and Pe, thus regression models (rather than mixed models) were used. Age was also included as a covariate in all analyses and its effects are not presented here for simplicity, but are available upon request.

ADHD-P, attention-deficit/hyperactivity disorder persisters; ADHD-R, attention-deficit/hyperactivity disorder remitters; Congruent, congruent condition; Ctrl, control; *d*, Cohen’s *d* effect size; ERN, error-related negativity; ERP, event-related potential; Incongruent, incongruent condition; MRT, mean reaction time of correct response to targets; *p*, regression model significant testing; Pe, error-related positivity; RTV, reaction time variability to targets (i.e., SD of reaction time).

a *p* ≤ .01.

b *d* > .80, indicating a large effect size.

c *d* > .50, indicating a medium effect size.

d *p* ≤ .09.

e *p* ≤ .05.

**Table 3:** Pearson Correlations (Two-Tailed) of Cognitive Performance and ERP Measures With Interview-Based DIVA ADHD Symptoms and Clinical Impairment Within the ADHD Group Only (*n* = 110)

	Controlling for Age and Sex		Controlling for IQ, Age, and Sex	
	ADHD Symptoms	Impairment	ADHD Symptoms	Impairment
Congruent Errors	.15	.21^a^	.10	.17^b^
Incongruent Errors	.07	.03	.05	<.01
Congruent MRT (ms)	−.11	<.01	.07	−.09
Incongruent MRT (ms)	.05	−.07	−.01	.14
Congruent RTV (ms)	.21^a^	.13	.15	.12
Incongruent RTV (ms)	.21^a^	.18^b^	.14	.10
N2 at Fz (µV)	.04	.18^b^	.04	.18^b^
N2 at FCz (µV)	.07	.12	.10	.15
ERN at FCz (µV)	−.01	−.15	.03	−.11
Pe at CPz (µV)	−.20^a^	−.20^a^	−.20^a^	−.20^a^

ADHD, attention-deficit/hyperactivity disorder; Congruent, congruent condition; DIVA, Diagnostic Interview for ADHD in adults; ERN, error-related negativity; ERP, event-related potential; Incongruent, incongruent condition; MRT, mean reaction time of correct response to targets; Pe, error-related positivity; RTV, reaction time variability to targets (i.e., SD of reaction time).

a *p* ≤ .05.

b *p* ≤ .09.
