# Supplementary material for: Attention-Deficit/Hyperactivity Disorder Remission Is Linked to Better Neurophysiological Error Detection and Attention-Vigilance Processes
Source: Biol Psychiatry. 2016 Dec 15;80(12):923–32. doi: 10.1016/j.biopsych.2016.06.021 (PMC5120985; doi:10.1016/j.biopsych.2016.06.021)
Supplement: Supplementary file 1 — Supplementary material [file mmc1.pdf]

Attention-Deficit/Hyperactivity Disorder Remission Is Linked to Better Neurophysiological Error Detection and Attention-Vigilance Processes

Supplement 1

Further Details on the Task

The version of Eriksen flanker task used in this study consisted of 10 blocks of 40 trials where congruent versus incongruent conditions and the direction of responses (left versus right) were counter-balanced and randomized. Participants were seated on an adjustable chair in an acoustically shielded, video-monitored room. Two practice blocks were administered before the task blocks and task comprehension was ascertained prior to task performance. On congruent trials, flanker and target arrowheads pointed in the same direction; on incongruent trials, they pointed in opposite directions. Flankers were presented every 1650 ms (inter-trial interval (ITI), therefore an inter-block interval (IBI) was 1400 ms). ITI and IBI were fixed. After each block, feedback was presented on screen to emphasize both speed and accuracy, in order to encourage participants to make enough errors to enable analysis of ERN/Pe components, and enough correct responses for analysis of the N2. Where participants made > 10% errors on congruent or > 40% errors on incongruent trials, they were instructed to slow down. Where participants made < 10% errors on congruent or < 40% errors on incongruent trials, they were instructed to perform faster. If neither rule applied, feedback informed participants to continue the same way. The task was run during a 1.5-hour recording session between two other tasks not reported here: preceded by the Continuous Performance Test-OX and followed by the Fast Task (see (1) for details). Breaks of at least 5 minutes were given in between tasks. Where necessary, participants were told to minimize movement or blinking.

Comparison Between Peak-to-Peak and Peak-to-Baseline ERN

In the present study the ERN was measured with reference to the previous preceding positive peak (the PNe, occurring between -100 and 50ms), consistently with several previous studies in the

literature (2-8). In these studies, this measurement of the ERN has proven useful to account for individual variability in amplitude range and to reduce latent low-frequency noise. The peak-to-peak ERN measure is also a robust index of early error processing, which has been shown to delineate ADHD from controls in independent samples using this version of the Eriksen flanker task (9-11). Since some previous studies on ADHD samples have instead measured the ERN as a peak-to-baseline measure (12-14), we also investigated the differences between ADHD remitters, persisters and controls on this peak-to-baseline measure and on the PNe directly, for completeness and to allow comparison with previous results.

For the peak-to-baseline ERN we found significant overall group differences ($p=.05$) and ADHD persisters vs. controls difference ($p<.01$), but ADHD remitters did not significantly differ from either persisters ($p=.47$) or controls ($p=.34$). We did not find any group differences on the PNe (overall group effect: $p=.18$; ADHD remitters vs. persisters: $p=.27$; ADHD remitters vs. controls: $p=.69$; ADHD persisters vs. controls: $p=.14$).

Considering these results, the peak-to-baseline ERN may be less sensitive to ADHD remission/persistence compared to the peak-to-peak ERN. Of note, the analysis of PNe alone showed no group differences on this measure, thus the inclusion of this earlier peak in the peak-to-peak measure of the ERN did not explain the group differences on the peak-to-peak ERN, which reflect the voltage change from the PNe to the negative ERN.

Table S1. Pearson correlations between cognitive and ERP measures and age, divided by group

	Pearson *r* with age		
	**Controls**	**ADHD-R**	**ADHD-P**
Congruent errors	0.01	-0.19	-0.23*
Incongruent errors	-0.14†	-0.27	-0.29*
Congruent MRT (ms)	0.13	0.02	0.17
Incongruent MRT (ms)	-0.15†	-0.02	-0.20†
Congruent RTV (ms)	0.18*	0.07	0.23*
Incongruent RTV (ms)	0.25**	0.05	0.18
N2 at Fz (µV)	-0.42**	-0.41†	-0.39*
N2 at FCz (µV)	-0.28**	-0.03	-0.15
ERN (peak) at FCz (µV)	-0.25**	0.19	-0.09
ERN (peak-to-peak) at FCz (µV)	-0.12	0.10	0.07
Pe at CPz (µV)	-0.36**	-0.46*	-0.25*

ADHD-R = ADHD remitters; ADHD-P = ADHD persisters; Congruent = congruent condition; Incongruent = incongruent condition; MRT = reaction time of correct response to targets; RTV = reaction time variability to targets (SD of reaction time).

**$p \leq .01$; *$p \leq .05$; †$p < .09$.

Table S2. Full results of mixed model analyses showing main effects of group, condition (congruency), site (for the N2 only), and interaction effects on cognitive-performance and ERP measures

	Group effect		Congruency effect		Group*Congruency		Site effect		Group*Site	
	z	p	z	p	z	p	z	p	z	p
Errors	7.95	<.01**	43.39	<.01**	-5.22	<.01**	-	-	-	-
MRT	0.16	.87	582.71	<.01**	2.91	<.01**	-	-	-	-
RTV	-10.24	<.01**	-90.07	<.01**	6.95	<.01**	-	-	-	-
N2	2.64	<0.01**	9.65	<.01**	-.33	.74	83.22	<0.01**	-3.36	<0.01**

MRT = reaction time of correct response to targets; RTV = reaction time variability to targets (SD of reaction time).

Data on performance measures were available for the full sample (87 ADHD-P, 23 ADHD-R and 169 controls); data on the N2 were available for 84 ADHD-P, 23 ADHD-R and 169 controls. Age was also included as a covariate and its effects not presented here for simplicity, but available on request. Only group effects were tested on the ERN and Pe, thus regression models (rather than mixed models) were used and the full results are presented in Table 2 of the main test.

**p≤.01; *p≤.05; †p<.09.

Table S3. Descriptive statistics and group comparison on cognitive-performance and ERP measures performed only on males

	ADHD-P	ADHD-R	Ctrl	Group Comparison						
	mean (SD)	mean (SD)	mean (SD)	p	ADHD-P vs Ctrl		ADHD-P vs ADHD-R		ADHD-R vs Ctrl	
					d	p	d	p	d	p
Performance										
Congruent errors	9.59 (13.570	4.00 (3.85)	4.01 (7.10)	<.01**	*.73*	<.01**	*.68*	.01**	.00	.97
Incongruent errors	57.96 (18.59)	56.22 (20.75)	50.66 (18.69)	<.01**	.46	<.01**	.09	.81	.35	.13
Congruent MRT (ms)	352.47 (62.91)	339.58 (38.99)	332.45 (33.08)	<.01**	.42	<.01**	.19	.43	.23	.32
Incongruent MRT (ms)	448.12 (59.19)	441.94 (33.44)	428.37 (41.89)	<.01**	.41	<.01**	.02	.95	.42	.02*
Congruent RTV (ms)	113.94 (70.72)	83.19 (28.22)	74.40 (19.75)	<.01**	**.99**	<.01**	*.53*	.01**	.43	.05*
Incongruent RTV (ms)	121.34 (86.84)	88.18 (32.91)	75.51 (22.99)	<.01**	**.97**	<.01**	.45	.05*	*.53*	.02*
ERPs										
N2 at Fz (µV)	-7.33 (3.74)	-6.91 (3.61)	-6.50 (3.30)	.02*	.34	.10	.05	.64	.29	.05*
N2 at FCz (µV)	-5.91 (3.89)	-6.26 (3.57)	-7.04 (3.92)	.09†	.26	.11	.14	.30	.12	.85

	ADHD-P	ADHD-R	Ctrl	Group Comparison						
	mean (SD)	mean (SD)	mean (SD)	p	ADHD-P vs Ctrl		ADHD-P vs ADHD-R		ADHD-R vs Ctrl	
					d	p	d	p	d	p
ERN at FCz (peak-to-peak, µV)	-7.97 (3.38)	-9.64 (4.11)	-9.99 (4.12)	<.01**	**.52**	<.01**	.47	.09†	.06	.78
Pe at CPz (µV)	9.67 (4.14)	10.96 (4.06)	11,50 (4.45)	.02*	.41	.02*	.38	.11	.04	.91

ADHD-P = ADHD persisters, ADHD-R = ADHD remitters, Ctrl = Control group, SD = standard deviation, p = regression model significant testing, d = Cohen's d effect size (0.2 small, 0.5 medium and 0.8 large), Congruent = congruent condition, Incongruent = incongruent condition, MRT = reaction time of correct response to targets, RTV = reaction time variability to targets (i.e. SD of reaction time).

Data on performance measures and N2 were available for 72 ADHD-P, 23 ADHD-R and 128 controls; data on the ERN and Pe were available for 69 ADHD-P, 19 ADHD-R and 111 controls.

Age was included as a covariate in all analyses and its effects are not presented here for simplicity, but available on requests.

Bold denotes a large effect size, italics denotes a medium effect size.

**p≤.01; *p≤.05; †p≤.09.

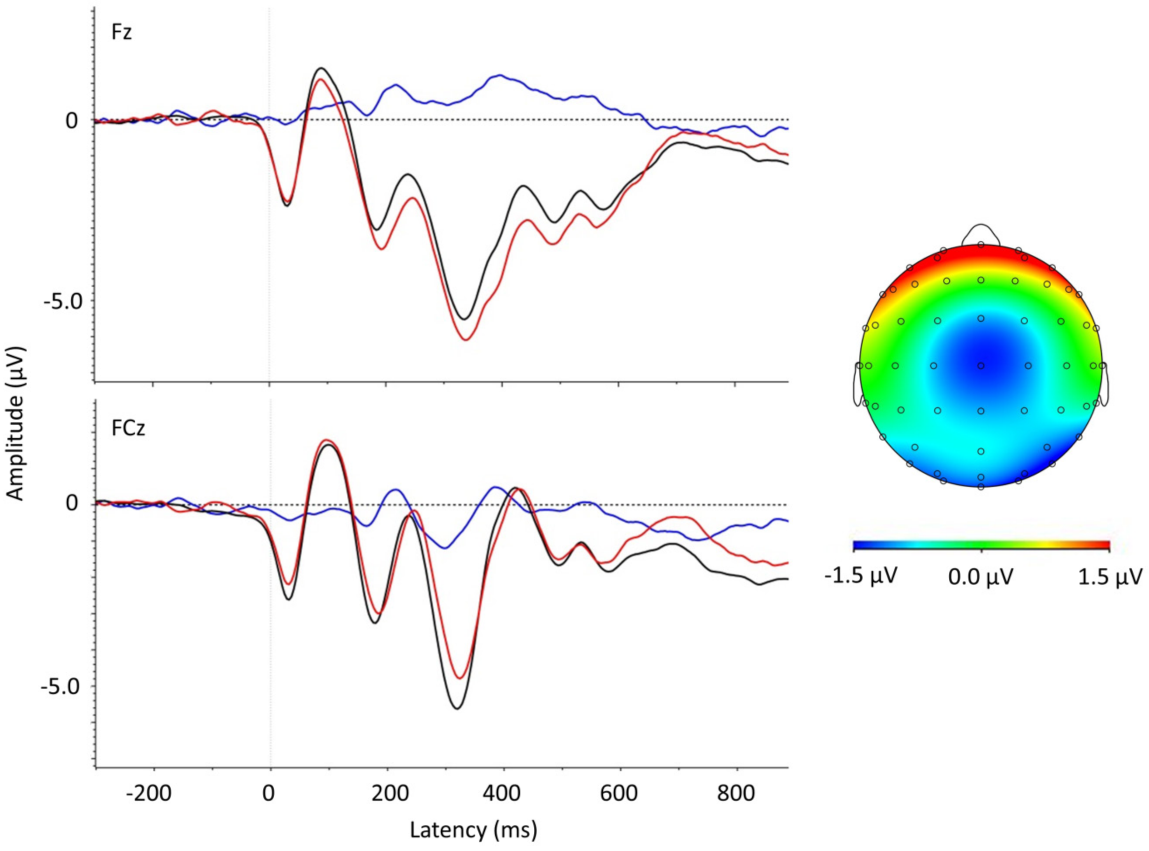

Figure S1. Difference wave (in blue) showing the difference between ADHD persisters (ADHD-P, in red) and control participants (Controls, in black) in grand average stimulus-locked ERPs of the N2 at Fz and FCz electrodes between 250-450 ms after incongruent stimuli where a correct response was made, with t-map. Topographical differences between ADHD persisters and controls can be observed here, which likely led to an enhanced N2 in persisters at Fz but with a trend for reduction at FCz. This is indicated in the grand average ERP showing the difference wave (in blue, ADHD persisters minus controls), which appears in the positive region of the graph at Fz (corresponding to the positive frontal difference in yellow-red in the t-map) and in the negative region of the graph at FCz (corresponding to the negative central difference in blue in the t-map).

Supplemental References

1. Cheung CH, Rijsdijk F, McLoughlin G, Brandeis D, Banaschewski T, Asherson P, et al. (2015): Cognitive and neurophysiological markers of ADHD persistence and remission. *Br J Psychiatry*.
2. Falkenstein M, Hoormann J, Christ S, Hohnsbein J (2000): ERP components on reaction errors and their functional significance: a tutorial. *Biol Psychol*. 51:87-107.
3. Falkenstein M, Hielscher H, Dziobek I, Schwarzenau P, Hoormann J, Sunderman B, et al. (2001): Action monitoring, error detection, and the basal ganglia: an ERP study. *Neuroreport*. 12:157-161.
4. Frank MJ, Woroch BS, Curran T (2005): Error-related negativity predicts reinforcement learning and conflict biases. *Neuron*. 47:495-501.
5. Gentsch A, Ullsperger P, Ullsperger M (2009): Dissociable medial frontal negativities from a common monitoring system for self- and externally caused failure of goal achievement. *Neuroimage*. 47:2023-2030.
6. Kopp B, Mattler U, Goertz R, Rist F (1996): N2, P3 and the lateralized readiness potential in a nogo task involving selective response priming. *Electroencephalogr Clin Neurophysiol*. 99:19-27.
7. Nieuwenhuis S, Ridderinkhof KR, Blom J, Band GP, Kok A (2001): Error-related brain potentials are differentially related to awareness of response errors: evidence from an antisaccade task. *Psychophysiology*. 38:752-760.
8. Nieuwenhuis S, Yeung N, van den Wildenberg W, Ridderinkhof KR (2003): Electrophysiological correlates of anterior cingulate function in a go/no-go task: effects of response conflict and trial type frequency. *Cogn Affect Behav Neurosci*. 3:17-26.
9. Albrecht B, Brandeis D, Uebel H, Heinrich H, Mueller UC, Hasselhorn M, et al. (2008): Action monitoring in boys with attention-deficit/hyperactivity disorder, their nonaffected siblings, and normal control subjects: evidence for an endophenotype. *Biol Psychiatry*. 64:615-625.
10. Albrecht B, Brandeis D, Uebel H, Heinrich H, Heise A, Hasselhorn M, et al. (2010): Action monitoring in children with or without a family history of ADHD--effects of gender on an endophenotype parameter. *Neuropsychologia*. 48:1171-1177.
11. McLoughlin G, Albrecht B, Banaschewski T, Rothenberger A, Brandeis D, Asherson P, et al. (2009): Performance monitoring is altered in adult ADHD: a familial event-related potential investigation. *Neuropsychologia*. 47:3134-3142.
12. Groom MJ, Scerif G, Liddle PF, Batty MJ, Liddle EB, Roberts KL, et al. (2010): Effects of motivation and medication on electrophysiological markers of response inhibition in children with attention-deficit/hyperactivity disorder. *Biol Psychiatry*. 67:624-631.
13. O'Connell RG, Bellgrove MA, Dockree PM, Lau A, Hester R, Garavan H, et al. (2009): The neural correlates of deficient error awareness in attention-deficit hyperactivity disorder (ADHD). *Neuropsychologia*. 47:1149-1159.
14. Wiersema JR, van der Meere JJ, Roeyers H (2009): ERP correlates of error monitoring in adult ADHD. *J Neural Transm*. 116:371-379.
